# Decision analytical modelling of strategies for investigating suspected acute aortic syndrome

**DOI:** 10.1136/emermed-2024-214222

**Published:** 2024-11-01

**Authors:** Praveen Thokala, Steve Goodacre, Graham Cooper, Robert Hinchliffe, Matthew J Reed, Steven Thomas, Sarah Wilson, Catherine Fowler, Valérie Lechene

**Affiliations:** 1 Division of Population Health, Sheffield Centre for Health and Related Research (SCHARR), The University of Sheffield Faculty of Medicine, Dentistry and Health, Sheffield, UK; 2 Cardiology and Cardiothoracic Surgery, Sheffield Teaching Hospitals NHS Foundation Trust, Sheffield, UK; 3 Department of Vascular Surgery, North Bristol NHS Trust, Westbury on Trym, UK; 4 Royal Infirmary of Edinburgh, Edinburgh, UK; 5 Academic Vascular Unit, Sheffield Teaching Hospitals, Sheffield, UK; 6 Emergency Department, Wexham Park Hospital, Slough, UK; 7 Aortic Dissection Charitable Trust, Chesterfield, UK

**Keywords:** cost efficiency

## Abstract

**Background:**

Acute aortic syndrome (AAS) requires urgent diagnosis with computed tomographic angiography (CTA). Diagnostic strategies need to weigh the benefits of detecting AAS against the costs of using CTA with a low yield of AAS when the prevalence of AAS is low. We aimed to estimate the cost-effectiveness of diagnostic strategies using clinical probability scoring and D-dimer to select patients with potential symptoms of AAS for CTA.

**Methods:**

We developed a decision analytical model to simulate the management of patients attending hospital with possible AAS. We modelled diagnostic strategies that used the Aortic Dissection Detection Risk Score (ADD-RS) and D-dimer to select patients for CTA. We used estimates from our meta-analysis, existing literature and clinical experts to model the consequences of diagnostic strategies on survival, health utility, and health and social care costs. We estimated the incremental cost per quality-adjusted life-years gained by each strategy compared with the next most effective alternative on the efficiency frontier.

**Results:**

A strategy based on the Canadian guideline (CTA if ADD-RS>1 or ADD-RS=1 with D-dimer >500 ng/mL) is cost-effective but would result in high rates of CTA if applied to an unselected population (AAS prevalence 0.26%). The strategy is also cost-effective and would result in lower rates of CTA if applied to a more selected population, such as those with a non-zero clinical suspicion of AAS (prevalence 0.61%). For patients currently receiving CTA, using ADD-RS>1 or D-dimer >500 ng/mL to select patients for CTA is cost-effective.

**Conclusions:**

A strategy using ADD-RS>1 or ADD-RS=1 with D-dimer >500 ng/mL to select patients for CTA appears cost-effective but primary research is required to evaluate this strategy in practice and determine how suspicion of AAS is identified.

**WHAT IS ALREADY KNOWN ON THIS TOPIC**

- Computed tomographic angiography (CTA) scanning of the aorta has high sensitivity and specificity for diagnosing acute aortic syndrome (AAS) but incurs significant costs and risks of ionising radiation. There are clinical scores and biomarkers available that can be used to select patients with suspected AAS for CTA.

**WHAT THIS STUDY ADDS**

- Our base case analyses showed that if strategies were applied unselectively to all patients with possible AAS, then CTA for those with Aortic Dissection Detection Risk Score (ADD-RS) >1 would be cost-effective at the £20 000 per quality-adjusted life-year (QALY) threshold but would result in more than half of cases of AAS being missed. CTA for those with ADD-RS>1 or ADD-RS=1 with D-dimer >500 ng/mL (modified Canadian guideline) would be cost-effective at the £30 000/QALY threshold but would require a threefold to fourfold increase in current CTA use, which may not be deliverable.

**HOW THIS STUDY MIGHT AFFECT RESEARCH, PRACTICE OR POLICY**

- A strategy based on the Canadian clinical practice guideline using ADD-RS>1 or ADD-RS=1 with D-dimer >500 ng/mL to select patients for CTA appears cost-effective but primary research is required to evaluate this strategy in practice and determine how suspicion of AAS is identified.

## Background

 Acute aortic syndrome (AAS) is a life-threatening emergency condition affecting the thoracic aorta. Without treatment, AAS can progress to aortic rupture, with rapid deterioration and death. AAS typically presents as chest pain, although back pain, abdominal pain and neurological deficits also occur. AAS incidence has been estimated at one in every 980 ED attendances with atraumatic chest pain,^1^ thus creating a substantial diagnostic challenge.

Computed tomographic angiography (CTA) scanning of the aorta has high sensitivity and specificity for diagnosing AAS but incurs significant costs and risks of ionising radiation. Clinical scores and biomarkers can be used to select patients with suspected AAS for CTA. The Aortic Dissection Detection Risk Score (ADD-RS), as outlined in table 1, uses high-risk conditions, pain features or examination features to identify patients at risk of AAS.^2^ The D-dimer blood test can be used to rule out AAS in patients with a low clinical probability of AAS.^3^ The ADD-RS and D-dimer are respectively the most extensively validated clinical score and biomarker for AAS.^4^

**Table 1:** The Aortic Dissection Detection Risk Score (ADD-RS)^2^

**High-risk conditions**	
• Marfan syndrome• Family history of aortic disease• Known aortic valve disease• Recent aortic manipulation• Known thoracic aortic aneurysm	1 point if any present
**High-risk pain features**	
Chest, back or abdominal pain described as:• Abrupt in onset• Severe in intensity• Ripping or tearing in quality	1 point if any present
**High-risk examinationfeatures**	
• Pulse deficit or systolic BP differential• Focal neurological deficit (with pain)• Murmur of aortic insufficiency (new, with pain)• Hypotension or shock state	1 point if any present

The ADD-RS is calculated on the presence of risk markers in the clinical categories of predisposing conditions, pain features and physical findings. The score allocates 1 point if the patient has a high-risk condition, 1 point if they have a high-risk symptom and 1 point if they have a high-risk examination finding, to give an overall score between 0 and 3. A threshold of greater than 0 or greater than 1 can then select patients for further investigation.

International guidelines vary in their recommendations for diagnostic assessment of suspected AAS.^5^ All recommend clinical probability assessment followed by CTA for high-risk patients, but Canadian guidelines recommend D-dimer for intermediate-risk patients,^6^ European Society for Cardiology guidelines recommend D-dimer for low-risk patients^7^ and American Heart Association guidelines do not identify a role for D-dimer.^8^ Determining an appropriate diagnostic strategy to select patients with suspected AAS for CTA involves weighing the benefits of using CTA to identify AAS against the harms and costs of unselective CTA use.

We aimed to develop a decision analytical model to estimate the cost-effectiveness (in terms of net benefit and incremental cost per quality-adjusted life-year (QALY) gained) of strategies using clinical probability scoring and D-dimer to select patients with suspected AAS for CTA and estimate the expected value of perfect information (EVPI) to highlight the amount healthcare decision makers could spend on future primary research.

## Methods

### Overview

We developed a decision analytical model in Microsoft Excel and applied diagnostic strategies to a hypothetical population of 1000 patients attending hospital with symptoms suggesting AAS. Cost-effectiveness of the diagnostic strategies, measured as the incremental cost per QALY gained by each strategy compared with the next most effective alternative, was estimated using a lifetime horizon and UK NHS healthcare perspective. Probabilistic analysis incorporated uncertainty in the parameter estimates to provide robust estimates of the mean costs and QALYs. Three online meetings were conducted to systematically source clinical input. The first workshop involved finalising the model specification and assumptions, the second workshop aimed to identify best sources of data for populating the model and the third workshop addressed key uncertainties in the modelling and input data. Slide decks developed based on relevant published literature were circulated prior to the meetings and included specific questions to be discussed at the workshops. Clinical experts (SG, GC, RH, MJR, ST, SW) ensured that the model reflected clinical practice and identified data sources to populate the model. Patient representatives (CF, VL) ensured that the analysis and interpretation of the findings took the patient perspective into account.

### Model structure and analysis

**Figure 1:**
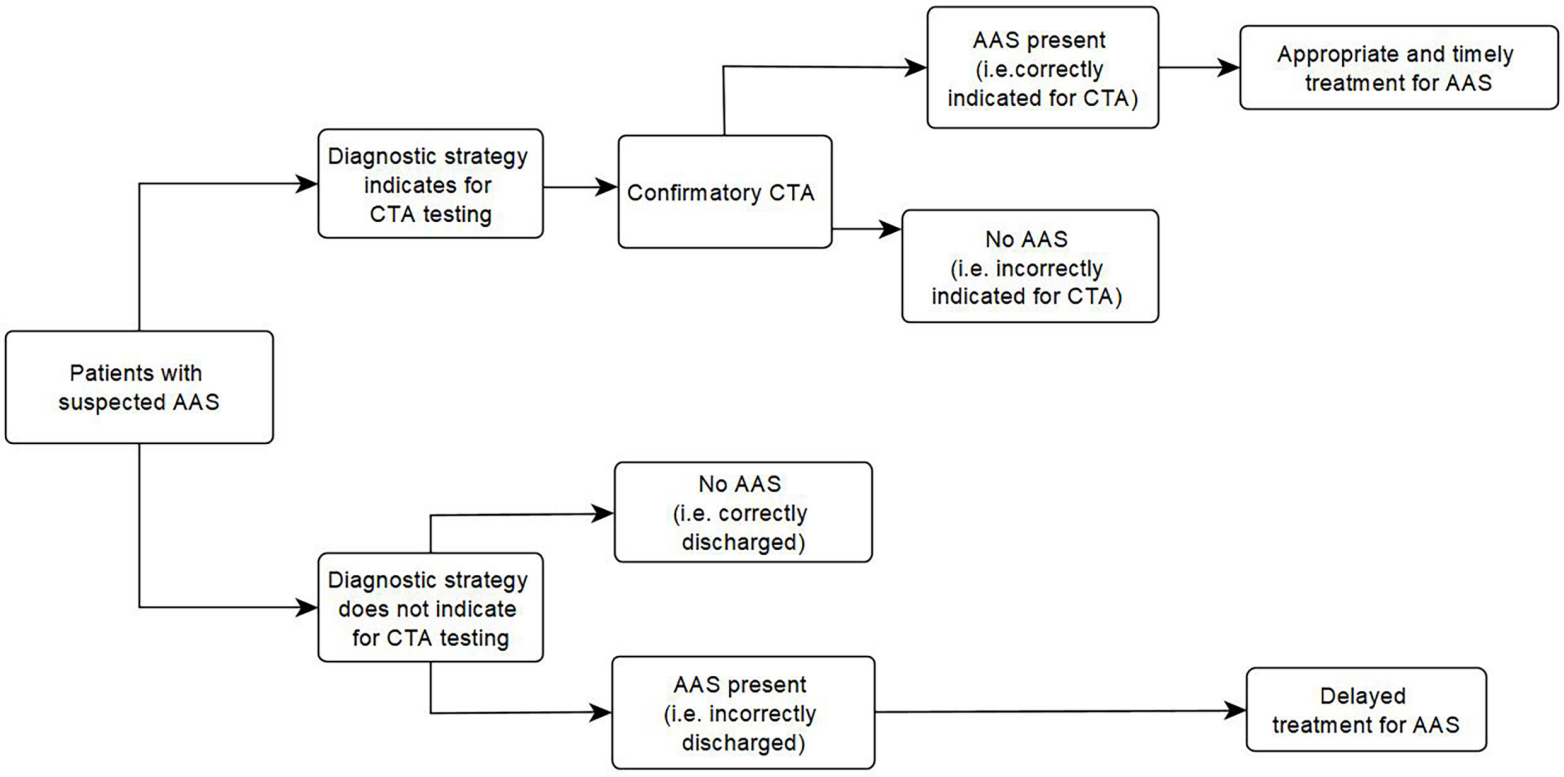
Model structure. AAS, acute aortic syndrome; CTA, computed tomographic angiography.

Figure 1 shows the structure of the model. Each diagnostic strategy was applied to the patient cohort to determine the proportions classified as true positive, false positive, true negative or false negative, depending on the prevalence of AAS and sensitivity or specificity of the diagnostic strategy. We assumed that all negatives would not receive further testing for AAS (including the false negatives, ie, those with AAS), and true positives and false positives would be identified after confirmatory testing using CTA as the reference standard for AAS (with false positives discharged and true positives receiving treatment for AAS). The QALYs for those without AAS were estimated based on life expectancy of general population from the Office of National Statistics, and the general population utilities estimated from Ara and Brazier.^9^ The model used half-cycle correction and used National Institute for Health and Care Excellence recommended discount rate of 3.5% per annum. Probabilistic sensitivity analyses were performed to capture the uncertainty in the model parameters. Uncertainty in the prevalence was parameterised using beta distributions based on the sample size of their respective studies.^10^ The outputs of the meta-analysis were used directly in the model to represent the uncertainty in the sensitivity and specificity of diagnostic strategies.^11^ For the other parameters, where there are not much data available to model the uncertainty, normal distributions were used and the SE was assumed to be 10% of the mean. For the utilities, the uncertainty was assumed to be 5% of the mean as there is less uncertainty around those estimates.

### Population

The characteristics of the hypothetical population were based on the Diagnosis of Acute Aortic Syndrome in the Emergency Department (DAShED) study, which recruited 5548 patients presenting to the ED with symptoms of possible AAS.^10^ It is unclear whether clinicians should apply diagnostic strategies to all patients with symptoms suggesting AAS or should exclude those at minimal risk to select a higher prevalence population. We therefore performed sensitivity analysis to explore this issue. The prevalence of AAS was estimated from the DAShED study,^10^ along with the mean age of the patients (63 years), while the proportion of type A and type B AAS and the proportion of males were estimated from the International Registry of Acute Aortic Dissection (IRAD) study, as shown in table 2.

**Table 2:** Prevalence of AAS and diagnostic accuracy and costs of the strategies tested in the model

Parameter	Value	Distribution	Source
**Epidemiology**			
Prevalence of AAS			
Primary analysis	0.26%	Beta (14, 5339)	DAShED study^10^
Secondary (AAS likelihood >0)	0.61%		
Secondary (AAS likelihood >1/10)	1.0%		
Secondary (AAS likelihood >2/10)	1.74%		
Secondary (currently receive CTA)	2.95%		
Proportion of type A patients	66.67%	Beta (2952, 1476)	IRAD^12^
Proportion of type B patients	33.33%	Beta (1476, 2952)	IRAD^12^
Proportion male	66.93%	Beta (2964, 1464)	IRAD^12^
**Diagnostic accuracy and costs of the strategies tested in the model** *			
	**Sensitivity**	**Specificity**	**Cost**
ADD-RS>1	41.6 (24.8, 59.1)	91.7 (81.7, 97)	£3.77
Modified Canadian guidelines (ADD-RS>1 or if ADD-RS=1 and D-dimer >500 ng/L)	93.1 (87.1, 96.3)	67.1 (54.4, 77.7)	£7.69
ADD-RS>0	95.1 (88.5, 98.4)	38 (20.1, 59.1)	£3.77
D-dimer >500 ng/mL	96.4 (94.9, 97.7)	56.6 (49.5, 63.4)	£7.30
ADD-RS>1 or D-dimer >500 ng/mL	98.3 (94.9, 99.5)	51.4 (38.7, 64.1)	£10.46
ADD-RS>0 or D-dimer >500 ng/mL	99.8 (98.7, 100)	21.8 (12.1, 32.6)	£6.54

*Estimated from meta-analysis

AASacute aortic syndromeADD-RSAortic Dissection Detection Risk ScoreCTAcomputed tomographic angiographyDAShEDDiagnosis of Acute Aortic Syndrome in the Emergency DepartmentIRADInternational Registry of Acute Aortic Dissection

The estimated prevalence of AAS was varied in sensitivity analysis to understand the impact of patient selection on cost-effectiveness. The primary analysis used the estimate of prevalence from the total population presenting with possible symptoms of AAS, that is, all patients presenting to the ED with new-onset chest, back or abdominal pain, syncope or symptoms related to malperfusion.^10^ Secondary analyses used prevalence estimates from populations selected on the basis of whether clinicians considered AAS as a possible diagnosis, based on the clinician’s estimate of the likelihood of AAS on a 0–10 scale, or were sufficiently concerned to order CTA, based on data from the DAShED study.^10^ We assumed that the population excluded patients whose frailty and/or comorbidities precluded surgery or thoracic endovascular aortic repair (TEVAR). These patients are implicitly excluded from diagnostic or outcome studies of AAS because they do not receive the relevant tests or treatments. Attempting to include them in our model would involve very limited data and would reduce the applicability of the model to the population most likely to benefit from investigation for AAS.

### Diagnostic strategies

We selected diagnostic strategies based on ADD-RS and/or D-dimer that reflected current guidelines and provided a range of trade-offs between sensitivity and specificity, estimated by meta-analysis of diagnostic cohort studies^11^ and summarised in table 2. The meta-analysis used data from six published studies that was shared by the authors to allow estimation of the sensitivity and specificity of each strategy. Full details are available in the open access publication.^11^ Along with these strategies, we also tested two hypothetical diagnostic strategies: no testing for AAS and CTA all. We assumed that no testing would have zero sensitivity and perfect specificity while CTA all, being the reference standard, would have perfect sensitivity and specificity.

The unit costs of ADD-RS and D-dimer were used to estimate the costs of different diagnostic strategies. For diagnostic strategies that combine both ADD-RS and D-dimer, it was assumed that ADD-RS would be performed first, and a proportion of patients would receive D-dimer (based on the specificity of the ADD-RS strategy).

### Short-term survival

We assumed that the diagnostic strategy only influenced outcomes among patients with AAS. The short-term survival depended on whether the condition was diagnosed and treated promptly, and whether the patients had type A or type B AAS, as presented in table 3.

**Table 3:** Mortality, cost and utility data used in the model

Parameter	Value	Distribution	Source
**Short-term survival of type A patients**			
Patients identified and treated surgically	80% at 2 months	Normal (0.8, 0.08)	IRAD^12^
Misdiagnosed type A patients	50% at 2 months	Normal (0.5, 0.05)	Matthews *et al*^13^ and Pourafkari *et al*^14^
**Short-term survival of type B patients**			
Type B patients identified promptly	87.4% at 2 months	Normal (0.87, 0.087)	Calculations
Misdiagnosed type B patients	74.8% at 6 months	Normal (0.748, 0.0748)	Calculations
**Annual mortality risk of survivors**			
Annual mortality risk of type A patients	2.5%	Normal (0.025, 0.0025)	IRAD^12^
Annual mortality risk of type B patients managed medically	5.5%	Normal (0.055, 0.0055)	Sá *et al*^16^
Annual mortality risk of type B patients receiving TEVAR	3.8%	Normal (0.038, 0.0038)	Sá *et al*^16^
**Annual probability of reintervention**			
Type A patients	0.77%	Beta (97, 2413)	Isselbacher *et al*^25^
Type B patients	1.62%	Beta (101, 1211)	Isselbacher *et al*^25^
**Utilities**			
Type A patients	0.792	Normal (0.792, 0.04)	Bojko *et al*,^21^ Ara and Brazier^23^
Type B patients medically managed	0.783	Normal (0.783, 0.039)	Meccanici *et al*,^22^ Ara and Brazier*l*^23^
Type B patients receiving TEVAR	0.862	Normal (0.862, 0.043)	Meccanici *et al*,^22^ Ara and Brazier^23^
Costs*			
Cost of CTA	£154.5	Normal (154.5, 15.45)	NHS reference costs^17^
Cost of CTA for incidental findings	£117	Normal (117, 11.70)	NHS reference costs^17^
Cost of D-dimer	£7.30	Normal (7.30, 0.73)	Cost of laboratory test (£6.79 in 2020 costs)
Cost of ADD-RS	£3.77	Normal (3.77, 0.377)	2 min of consultant time
Costs of open repair	£34 553	Normal (34 553, 3455)	NHS reference costs^17^
Cost of TEVAR	£13 973	Normal (13 973, 1397)	NHS reference costs^17^
Costs of medical management for type B patients (first year)	£4887.70	Normal (4887.7, 488.70)	NHS reference costs^17^
Annual costs for AAS survivors who received thoracic endovascular aortic repair (TEVAR) or medical management	£411.20	Normal (411.2, 41.12)	NHS reference costs^17^
Annual costs of AAS survivors who received open surgery	£517.78	Normal (517.78, 51.78)	NHS reference costs^17^
Costs of ED death	£885.27	Normal (885.27, 88.52)	NHS reference costs^17^
**Cancer due to CTA**			
Risk of cancer due to CTA	0.15%	Normal (0.0015, 0.00015)	Huang *et al*^18^
Costs of cancer†	£18 248.57	Normal (18 248.57, 1824.86)	Goodacre *et al*^19^
QALY loss due to cancer†	−0.12	−Normal (0.12, 0.006)	Goodacre *et al*^19^

* See appendix A2online supplemental appendix A2 for details.

† aApplied at 12 years, that is, mid-point of life expectancy.

AASacute aortic syndromeADD-RSAortic Dissection Detection Risk ScoreCTAcomputed tomographic angiographyIRADInternational Registry of Acute Aortic DissectionQALYquality-adjusted life-year

In the model, it was assumed that all patients with type A AAS who were diagnosed promptly (ie, during the initial ED visit) would receive surgical treatment, and their survival was estimated as 80% at 2 months based on IRAD data.^12^ Patients with type A AAS who were misdiagnosed and discharged were assumed to die without treatment or have delayed diagnosis and treatment, with survival estimated as 50% at 2 months based on studies by Matthews *et al*,^13^ and Pourafkari *et al*,^14^ who report the impact of delayed treatment on survival.

Among patients with type B AAS who were diagnosed promptly, it was assumed that 10% received surgical treatment, with the rest under medical management. Their respective short-term survival was estimated from IRAD data^12^ (see online supplemental appendix A1 for details), resulting in an overall survival of 87.4% for patients with type B AAS at 2 months. Patients with type B AAS who were misdiagnosed were assumed to forego the benefit of BP management, resulting in 2-month survival of 74.8%, based on the relative risk for BP control.^15^

### Long-term survival

The annual mortality risk for patients with type A AAS was estimated as 2.5% based on IRAD data.^12^ For patients with type B AAS, the annual mortality risk was 5.5% for those who are managed medically, and 3.8% for those receiving TEVAR, based on data from Sá *et al*.^16^ These risks were converted into standardised mortality ratios and applied in the model (see online supplemental appendix A1 for details).

We assumed that patients who were misdiagnosed during initial investigation would die or their AAS would be identified at some point in the following year. As such, a higher risk of mortality was applied to survivors for the first 6 months (ie, midpoint of the first year) using the same relative risks as those applied in the first 2 months (see online supplemental appendix A1), respectively.

### Costs

The model included costs of diagnostic strategies, CTA, CT for incidental findings, treatment for AAS, costs of death in ED and long-term costs associated with AAS management as presented in table 3. The costs of D-dimer were based on laboratory test costs, and the costs of ADD-RS were based assuming 2 min of consultant time. The costs of CTA, open repair and TEVAR were estimated from the NHS reference costs,^17^ while the costs of medical management for type B AAS and AAS survivors were estimated using expert clinical opinion. We used a 5% probability of incidental findings to estimate the additional costs of CT scans for incidental findings, based on estimates from the clinical experts who drew on audit data from their hospitals. We assumed that patients who were misdiagnosed at initial investigation but had AAS subsequently identified in the following year would incur the costs of treatment for AAS at 6 months.

In the long term, all survivors had an annual probability of reintervention which was estimated based on IRAD data, and it was assumed that all reinterventions are TEVAR. The model also included a small risk of cancer associated with CTA based on study by Huang *et al*^18^ and modelled the impact of cancer as one-off lifetime cost and QALY loss estimated from Goodacre *et al*^19^ at the midpoint of life expectancy estimated from UK life tables.

### Utilities

The utilities for patients with AAS were sourced from studies identified in the recent systematic review by Carbone *et al*.^20^ For patients with type A AAS, Bojko *et al*^21^ report the eight Short-Form Six-Dimension (SF-6D) dimension scores, and for patients with type B AAS, Meccanici *et al*^22^ report the eight mean SF-6D dimension scores of patients receiving TEVAR and medical management, respectively. These mean SF-6D dimension scores were converted into mean EuroQoL Five-Dimension (EQ-5D) utilities as reported in table 3, using an algorithm developed by Ara and Brazier.^23^ In the model, the patient utilities were capped at the age-specific general population utilities to ensure the patient utilities do not exceed the utilities of the general population.

### Patient and public involvement

Two members of the study team provided a patient and public perspective. VL has experienced AAS and CF is a relative of a patient who died from AAS. Their roles in the study are outlined in the author contributions. Also, the model was presented to a patient and public representative group involving members of the Aortic Dissection Charitable Trust (https://aorticdissectioncharitabletrust.org/), who provided feedback on the model assumptions and initial findings.

## Results

Table 4 shows the deterministic cost-effectiveness results for the base case analysis, as well as the number of CTAs performed and number of cases of AAS detected and missed at a typical hospital (with 3281 cases of possible AAS per year based on extrapolating from the incidence of cases in the DAShED study^10^). CTA for those with ADD-RS>1 was cost-effective at the £20 000/QALY threshold, but over half of cases of AAS would be missed. CTA for those with ADD-RS>1 or ADD-RS=1 with D-dimer >500 ng/mL was cost-effective at the £30 000/QALY threshold, but this would require CTA capacity to be increased threefold or fourfold, compared with an estimate of 298 per year extrapolated from the DAShED study.^10^

**Table 4:** Base case cost-effectiveness results and results for a typical hospital (AAS prevalence 0.26%)

	Cost-effectiveness results*			Typical hospitalNumber of suspected AAS=3281		
	**Total costs**	**Total QALYs**	**ICER(cost/QALY gained)**	**Number of CTAs**	**Number of cases of AAS detected** †	**Number of cases of AAS missed** ‡
CTA all	£255.62	11.10741	Dominated	3289.53	8.53	0.00
ADD-RS>0 or D-dimer >500 ng/mL	£222.88	11.10743	£1 812 565	2567.58	8.51	0.02
ADD-RS>1 or D-dimer >500 ng/mL	£173.56	11.10741	£137 338	1598.81	8.39	0.15
D-dimer >500 ng/mL	£160.46	11.10732	Extendedly dominated	1428.48	8.22	0.31
ADD-RS>0	£189.58	11.10722	Extendedly dominated	2037.04	8.11	0.42
ADD-RS>1 or ADD-RS=1 with D-dimer >500	£140.97	11.10717	£25 789	1084.58	7.94	0.59
ADD-RS>1	£74.81	11.10460	£15 990	275.16	3.55	4.98
No testing or CTA	£41.37	11.10251	–	0.00	0.00	8.53

* Cost-effectiveness results estimated as the average of patient cohort.

† Number of cases of AAS estimated as *Number of cases=Number of suspected AAS×Prevalence of AAS.*

‡ Number of cases of AAS missed estimated as *Number of AAS cases missed=Number of cases of AAS×(1-sensitivity).*

AASacute aortic syndromeADD-RSAortic Dissection Detection Risk ScoreCTAcomputed tomographic angiographyICERincremental cost-effectiveness ratioQALYquality-adjusted life-year

**Figure 2:**
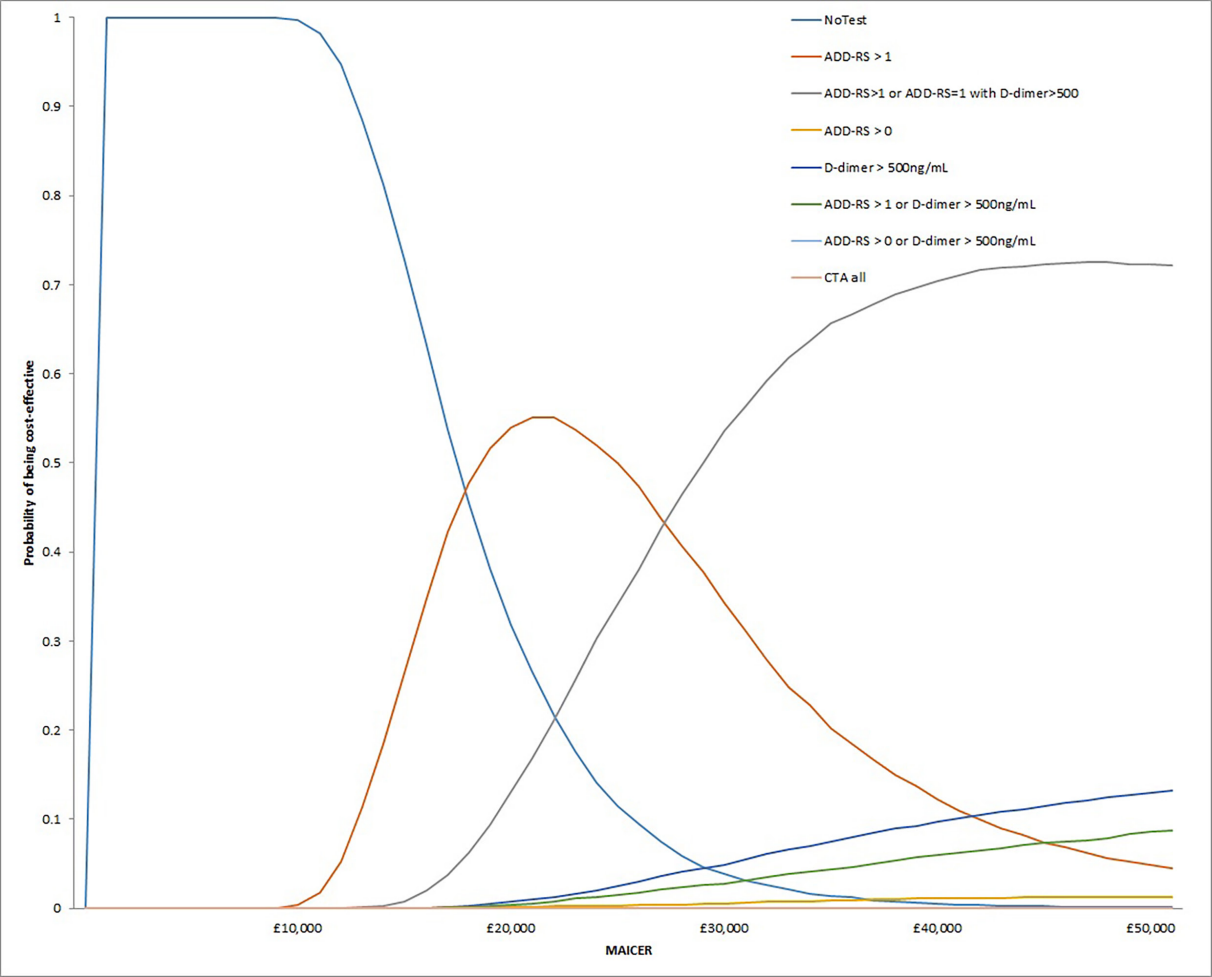
Cost-effectiveness acceptability curves from the base case probabilistic sensitivity analysis. ADD-RS, Aortic Dissection Detection Risk Score; CTA, computed tomographic angiography; MAICER, maximum acceptable incremental cost-effectiveness ratio.

Figure 2 shows the cost-effectiveness acceptability curves from the base case probabilistic sensitivity analysis. It shows that as the maximum acceptable incremental cost-effectiveness ratio increases, the strategy with the greatest probability of being cost-effective changes from no testing to ADD-RS>1 and then ADD-RS>1 or ADD-RS=1 with D-dimer >500 ng/mL. The EVPI analysis showed that at a threshold of £20 000/QALY, using a population size of 796 538 patients with suspected AAS to the NHS each year and a 5-year horizon, the individual EVPI was £4.46 per patient and the population EVPI was £17.75 million.

Table 5 shows the results of secondary analysis assuming 0.61% and 2.95% prevalence of AAS. Results of the sensitivity analyses assuming 1% and 1.74% prevalence are presented in online supplemental appendix A3. The 0.61% prevalence corresponds to the analysis in the DAShED cohort when patients with a clinician-estimated probability of AAS of zero were excluded. If clinicians are able to use their judgement to exclude patients they consider to have zero risk of AAS, then the modified Canadian guideline (CTA for those with ADD-RS>1 or with ADD-RS=1 and D-dimer >500 ng/mL) is cost-effective at the £20 000/QALY and £30 000/QALY thresholds, and would require 465 CTAs per year in a typical hospital (1.56-fold increase). The 2.95% prevalence of AAS relates to analysis in the DAShED study of the cohort who received CTA. This suggests that CTA for all is not cost-effective compared with selective strategies using ADD-RS and D-dimer. CTA for those with ADD-RS>1 or D-dimer >500 ng/mL is cost-effective at the £20 000/QALY or £30 000/QALY threshold and would approximately halve the number of CTAs required compared with CTA all.

**Table 5:** Secondary cost-effectiveness results (AAS prevalence 0.61% and 2.95%)

	Cost-effectiveness results* (AAS prevalence 0.61%)				Cost-effectiveness results (AAS prevalence 2.95%)			
	**Total costs**	**Total QALYs**	**ICER(cost/QALY gained)**	**Number of CTAs (typical hospital)**	**Total costs**	**Total QALYs**	**ICER(cost/QALY gained)**	**Number of CTAs (typical hospital)**
CTA all	£359.43	11.09010	Dominated	1406.53	1053.49	10.97441	£457 862	297.53
ADD-RS>0 or D-dimer >500 ng/mL	£326.19	11.09011	£384 837	1095.08	£1016.89	10.97433	£65 685	227.84
ADD-RS>1 or D-dimer >500 ng/mL	£276.33	11.08998	£59 159	683.67	£963.44	10.97351	£17 262	144.69
D-dimer >500 ng/mL	£262.35	11.08977	Extendedly dominated	611.25	£943.55	10.97243	Extendedly dominated	129.94
ADD-RS>0	£290.72	11.08958	Extendedly dominated	869.58	£966.96	10.97165	Extendedly dominated	182.00
ADD-RS>1 or ADD-RS=1 with D-dimer >500	£241.34	11.08939	£14 955	465.08	£912.34	10.97055	£8662	100.21
ADD-RS>1	£150.51	11.08332	£10 851	118.87	£656.58	10.94103	£7841	26.83
No testing or CTA	£97.07	11.07840	–	0.00	£469.43	10.91716	–	0.00

* cCost-effectiveness results estimated as the average of patient cohort .

.ADD-RS, Aortic Dissection Detection Risk ScoreCTAcomputed tomographic angiographyICERincremental cost-effectiveness ratioQALYquality-adjusted life-year

## Discussion

Our decision analytical modelling is based on robust estimates from our meta-analysis of the accuracy of the strategies and draws on clinical expertise to ensure a model that reflects the complexities of the clinical problem while retaining transparency. Our base case analyses showed that if strategies were applied unselectively to all patients with possible AAS, then CTA for those with ADD-RS>1 would be cost-effective at the £20 000/QALY threshold but would result in more than half of cases of AAS being missed. This reflects the low prevalence of AAS for those with ADD-RS≤1 in the base case analysis. The costs of investigating for AAS do not justify the benefits when the prevalence is very low. CTA for those with ADD-RS>1 or ADD-RS=1 with D-dimer >500 ng/mL (modified Canadian guideline) would be cost-effective at the £30 000/QALY threshold but would require a threefold to fourfold increase in current CTA use, which may not be deliverable.

Our base case analysis assumed that clinicians would apply the strategies unselectively to all patients with possible symptoms of AAS. However, the DAShED study showed that clinicians consider most of these patients to have zero likelihood of AAS. Our sensitivity analyses showed that if clinicians were to use their judgement to exclude patients considered to have a zero risk of AAS (resulting in a prevalence of 0.61%), then the strategy of CTA for patients with ADD-RS>1 or ADD-RS=1 with D-dimer >500 ng/mL would be cost-effective and require a more modest (1.56×) increase in CTA capacity. A strategy of ADD-RS>1 or D-dimer >500 ng/mL is cost-effective at 2.95% prevalence of AAS, which is the prevalence of AAS in patients receiving CTA in the DAShED study, and could thus offer a cost-effective alternative for those currently receiving CTA.

In a previous study, Taylor and Iyer^24^ used decision analytical modelling to compare testing strategies for AAS in terms of health outcomes but not costs. Their model suggested that low testing thresholds of 0.03% probability of AAS for CTA and 0.013% for D-dimer compared with no testing should be used. These findings suggest that the benefits of accurate diagnosis substantially outweigh the risks of testing, but do not take costs into account. Our analysis suggests that CTA for all is not cost-effective compared with alternative strategies when costs are considered rather than health outcomes alone. Furthermore, our base case analysis showed that even if strategies are cost-effective, they may require an increase in CTA capacity that may not be deliverable in a typical hospital.

A key limitation in our understanding of AAS diagnosis relates to how clinicians determine whether a patient presenting with symptoms compatible with AAS is considered to require investigation for AAS. Our base case analysis included all patients whose symptoms were compatible with AAS. However, the DAShED study^10^ showed that clinicians considered a substantial proportion of these patients to have a low or zero likelihood of AAS and only used CTA to investigate a minority for AAS. Our analysis showed that strategies involving the ADD-RS and D-dimer are cost-effective and more likely to be deliverable if limited to selected patients in whom clinical judgement suggests a meaningful risk of AAS. The limitation with this finding is that we do not know how clinicians make this judgement and whether their assessment of zero AAS risk is accurate.

A related limitation is that we did not compare the strategies to using unstructured clinical judgement to select patients for CTA or consider how clinical judgement could be used alongside the strategies. If we conclude that clinical judgement is required to select patients for diagnostic investigation with the ADD-RS and D-dimer, then we should consider whether the selection for CTA should be based on clinical judgement alone (or informed by ADD-RS and D-dimer). There is little evidence available to estimate the accuracy of unstructured clinical judgement for diagnosing AAS, so we were unable to evaluate it in our analysis.

It is also important to note that we assumed that the population for the modelling excluded patients whose frailty and/or comorbidities meant that they would not be eligible for surgery or endovascular repair if AAS were detected. We made this assumption because these patients are implicitly excluded from studies of tests or treatments for AAS. Including them in the model would involve multiple assumptions based on limited data, and would reduce the applicability of findings to the population most likely to benefit from AAS diagnosis. We felt that this assumption reflected clinical practice, which involves assessing the potential implications for treatment before ordering a diagnostic test. Our findings are therefore not applicable to patients with frailty or comorbidities that limit the treatment options to medical treatment alone. The decision to investigate such patients is likely to be individualised and involve consideration (and discussion with the patients) of whether investigation is in the patient’s best interests. Our clinical experts indicated that surgery or TEVAR would be unusual for patients with a clinical frailty score above 6 and decision-making would consider the risk factors for adverse outcome after surgery or TEVAR outlined in international guidelines.^7^

Other limitations relate to uncertainties in the assumptions and estimates used in the model. We used estimates of the effect of delayed treatment that are inevitably based on limited observational data. We were also unable to include any credible estimates of the benefits and harms arising from non-AAS diagnoses and incidental findings identified on CTA due to the variety of findings identified and uncertainty over their clinical significance. The 5% rate of repeat CT for incidental findings is also subject to uncertainty and may be an underestimate. Finally, we assumed that D-dimer results could be provided without significant delay and that false positive D-dimer results would not require further investigation after negative CTA. If results take several hours to deliver, then delayed diagnosis could lead to harm and undermine the cost-effectiveness of strategies involving D-dimer. If false positive results incur additional testing, the cost-effectiveness may also be undermined.

Our value of information analyses estimate that at a threshold of £20 000/QALY, the individual EVPI was £4.46 per patient and the population EVPI for patients presenting to the NHS with suspected AAS over 5 years was estimated as £17.75 million, suggesting that further research to reduce the uncertainty would be valuable.

In conclusion, a strategy based on the Canadian clinical practice guideline using ADD-RS>1 or ADD-RS=1 with D-dimer >500 ng/mL to select patients for CTA appears cost-effective but primary research is required to evaluate this strategy in practice and determine how suspicion of AAS is identified.

## supplementary material

10.1136/emermed-2024-214222 online supplemental file 1

10.1136/emermed-2024-214222 online supplemental file 2

10.1136/emermed-2024-214222 online supplemental file 3

## Data Availability

All data relevant to the study are included in the article or uploaded as supplementary information.
