# Supplementary material for: Decision analytical modelling of strategies for investigating suspected acute aortic syndrome
Source: Emerg Med J. 2024 Nov 1;41(12):e214222. doi: 10.1136/emermed-2024-214222 (PMC11671881; doi:10.1136/emermed-2024-214222)
Supplement: online supplemental file 1 [file emermed-41-12-s001.pdf]

Appendix A1: Survival estimates and utilities used in the modelling

Parameter	Value	Distribution	Source
Short-term survival of Type A patients			
Patients identified and treated surgically	80% at 2 months	Normal (0.8, 0.08)	IRAD[11]
Misdiagnosed Type A patients	50% at 2 months	Normal (0.5, 0.05)	Matthews et al.,[12] and Pourafkari et al.[13],
Short-term survival of Type B patients			
Patients identified and treated with TEVAR	91% at 2 months	Normal (0.91, 0.091)	IRAD[11]
Patients identified and managed medically	87% at 2 months	Normal (0.87, 0.087)	IRAD[11]
Proportion receiving TEVAR	10%	Normal (0.1, 0.01)	Personal communication
Type B patients identified promptly	87.4% at 2 months	Normal (0.87, 0.087)	Calculations
Relative risk of misdiagnosed patients	2.0	Normal (2, 0.2)	Nejim et al[14]
Misdiagnosed Type B patients	74.8% at 6 months	Normal (0.748, 0.0748)	Calculations
Annual mortality risk of survivors			
Annual mortality risk of Type A patients	2.5%	Normal (0.025, 0.0025)	IRAD[11]
Annual mortality risk of Type B patients managed medically	5.5%	Normal (0.055, 0.0055)	Sa et al[15]
Annual mortality risk of Type B patients receiving TEVAR	3.8%	Normal (0.038, 0.0038)	Sa et al[15]
Annual probability of re-intervention			
Type A patients	0.77%	Beta (97,2413)	Isselbacher et al 2016[16]
Type B patients	1.62%	Beta (101,1211)	Isselbacher et al 2016[16]
**Utilities**			

Type A patients	0.792	Normal (0.792, 0.04)	Bojko et al[17], Ara et al[18]
Type B patients medically managed	0.783	Normal (0.783, 0.039)	Meccanici et al 2023[19] , Ara et al[18]
Type B patients receiving TEVAR	0.862	Normal (0.862, 0.043)	Meccanici et al 2023[19], Ara et al[18]

ᵻ See appendix A2 for details

*applied at 12 years i.e. mid-point of life expectancy
