# Supplementary material for: Decision analytical modelling of strategies for investigating suspected acute aortic syndrome
Source: Emerg Med J. 2024 Nov 1;41(12):e214222. doi: 10.1136/emermed-2024-214222 (PMC11671881; doi:10.1136/emermed-2024-214222)
Supplement: online supplemental file 2 [file emermed-41-12-s002.pdf]

Appendix A2: Details of the costs used in the model

**Costs**			
Cost of CTA	£154.5	Normal (154.5, 15.45)	NHS reference costs[20]<br>Weighted average of Computerised Tomography Scan of Two, Three or More Areas, with Contrast<br>(RD24Z, RD26Z and RD27Z)
Cost of CT for incidental findings	£117	Normal (117, 11.70)	NHS reference costs[20] Computerised Tomography Scan of One Area, without Contrast (RD20A)
Cost of D-dimer	£7.30	Normal (7.30, 0.73)	Cost of lab test (£6.79 in 2020 costs)
Cost of ADD-RS	£3.77	Normal (3.77, 0.377)	2 minutes of consultant time
Costs of open repair	£34,553	Normal (34553, 3455)	NHS reference costs[20]<br>Open Repair of Thoracoabdominal Aortic Aneurysm (YQ06Z)
Cost of TEVAR	£13,973	Normal (13973, 1397)	NHS reference costs[20]<br>Weighted average of Complex and Standard Endovascular Repair of Thoracic Aortic Aneurysm, with CC Score 6+ (YR64A and YR65A)
Costs of medical management for Type B patients (first year)	£4,887.70	Normal (4887.7, 488.70)	NHS reference costs[20]<br>See Appendix A2 for details

Annual costs for AAS survivors who received TEVAR or medical management	£411.20	Normal (411.2, 41.12)	NHS reference costs[20] See Appendix A2 for details
Annual costs of AAS survivors who received open surgery	£517.78	Normal (517.78, 51.78)	NHS reference costs[20] See Appendix A2 for details
Costs of ED death	£885.27	Normal (885.27, 88.52)	NHS reference costs[20] Emergency Medicine, Any Investigation with Category 5 Treatment (VB01Z)
**Cancer due to CTA**			
Risk of cancer due to CTA	0.15%	Normal (0.0015, 0.00015)	Huang et al[21]
Costs of cancer*	£18,248.57	Normal (18248.57, 1824.86)	Goodacre et al[22]
QALY loss due to cancer*	-0.12	-Normal (0.12, 0.006)	Goodacre et al[22]

¥Estimated from meta-analysis

Cost of medical management for Type B AAS patients

	Details	Unit Cost
Costs of additional CTA	4 CTA in a year (30 days, 3 months, 6 months, 9 months)	£154.5
HDU/ICU stay	2 days in HDU/ICU	£1128.5 (assumed to be average of HDU/ICU costs)
General ward stay	4 days in general ward	£402
Nurse visits	4 nurse visits in a year	£20 using nurse cost of £51 per hour (average of bands 4–8b)
Outpatient reviews	2 outpatient reviews in year	£148

Beta-blocker	Atenolol 50mg daily or Bisoprolol 5mg daily	£0.98 for 28 tablets
Ace inhibitor	Ramipril 10mg daily	£1.22 for 28 tablets
Type B AAS medical management costs (in the first year)		£4887.70

Annual costs for AAS patients who received TEVAR or medical management

	Details	Unit Cost
Costs of additional CTA	1 CTA in a year	£154.5
Nurse visits	4 nurse visits in a year	£20 using nurse cost of £51 per hour (average of bands 4–8b)
Outpatient review	1 outpatient review in year	£148
Beta-blocker	Atenolol 50mg daily	£0.98 for 28 tablets
Ace inhibitor	Ramipril 10mg daily	£1.22 for 28 tablets
Annual costs (beyond first year)		£411.20

Annual costs for AAS patients who received open surgery

	Details	Unit Cost
Costs of MRI	1 CTA in a year	£261.12
Nurse visits	4 nurse visits in a year	£20 using nurse cost of £51 per hour (average of bands 4–8b)
Outpatient review	1 outpatient review in year	£148
Beta-blocker	Atenolol 50mg daily	£0.98 for 28 tablets
Ace inhibitor	Ramipril 10mg daily	£1.22 for 28 tablets
Annual costs (beyond first year)		£517.78
