# Supplementary material for: Decision analytical modelling of strategies for investigating suspected acute aortic syndrome
Source: Emerg Med J. 2024 Nov 1;41(12):e214222. doi: 10.1136/emermed-2024-214222 (PMC11671881; doi:10.1136/emermed-2024-214222)
Supplement: online supplemental file 3 [file emermed-41-12-s003.pdf]

Appendix A3: Results using an AAS prevalence of 1% and 1.74%

Secondary cost-effectiveness results (AAS prevalence 1%)

	**Cost-effectiveness results***			**Typical Hospital Number of suspected AAS = 853**		
	**Total Costs**	**Total QALYs**	**ICER (Cost/QALY gained)**	**# of CTA**	**# cases of AAS detected**	**# cases of AAS missed**
CTA all	£475.11	11.07082	10,154,402	861.53	8.53	0.00
ADD-RS > 0 or D-dimer > 500ng/mL	£441.31	11.07082	£207,134	668.89	8.51	0.02
ADD-RS > 1 or D-dimer > 500ng/mL	£390.85	11.07057	£38,202	418.80	8.38	0.15
D-dimer > 500ng/mL	£375.88	11.07021	Extendedly dominated	374.72	8.22	0.31
ADD-RS > 0	£403.43	11.06993	Extendedly dominated	531.68	8.11	0.42
ADD-RS>1 or ADD-RS=1 with D-dimer>500	£353.17	11.06959	£11,853	285.77	7.94	0.59
ADD-RS > 1	£234.85	11.05960	£9,369	73.64	3.55	4.98
No Testing or CTA	£159.13	11.05152	-	0.00	0.00	8.53

Secondary cost-effectiveness results (AAS prevalence 1.74%)

	**Cost-effectiveness results***			**Typical Hospital Number of suspected AAS = 490**		
	**Total Costs**	**Total QALYs**	**ICER (Cost/QALY gained)**	**# of CTA**	**# cases of AAS detected**	**# cases of AAS missed**
CTA all	£694.60	11.03423	£1,076,140	498.53	8.53	0.00
ADD-RS > 0 or D-dimer > 500ng/mL	£659.73	11.03420	£112,167	385.02	8.51	0.02
ADD-RS > 1 or D-dimer > 500ng/mL	£608.14	11.03374	£24,647	242.38	8.38	0.14

D-dimer > 500ng/mL	£591.31	11.03310	Extendedly dominated	217.18	8.22	0.31
ADD-RS > 0	£617.28	11.03263	Extendedly dominated	306.62	8.11	0.42
ADD-RS>1 or ADD-RS=1 with D-dimer>500	£565.37	11.03200	£9,798	166.34	7.94	0.59
ADD-RS > 1	£394.89	11.01460	£8,385	43.51	3.55	4.98
No Testing or CTA	£276.89	11.00053	-	0.00	0.00	8.53

*cost-effectiveness results estimated as the average of patient cohort

ADD-RS: Aortic Dissection Detection Risk Score; CTA, computer tomographic angiography; ICER, incremental cost-effectiveness ratio; QALY, quality-adjusted life year
